# Markers for Risk of Type 1 Diabetes in Relatives of Alsacian Patients With Type 1 Diabetes

**DOI:** 10.1080/15604280212527

**Published:** 2002

**Authors:** Jean Claude Ongagna, Remi Sapin, Michel Pinget, Alain Belcourt

**Affiliations:** 1 Centre Européen d'Etude du Diabète-Hôpitaux Universitaires de Strasbourg France; 2 Institut de Physique Biologique Unité d'Analyses Endocriniennes UPRES-A 7004-ULP/CNRS Faculté de Médecine Université Louis Pasteur de Strasbourg France

## Abstract

*Background*: The cytotoxic T lymphocyteassociated antigen 4 gene (CTLA-4) encode the T cell receptor involved in the control of T cell proliferation and mediates T cell apoptosis. The receptor protein is a specific T lymphocyte surface antigen that is detected on cells only after antigen presentation. Thus, CTLA-4 is directly involved in both immune and autoimmune responses and may be involved in the pathogenesis of multiple T cell-mediated autoimmune disorders. There is polymorphism at position 49 in exon 1 of the CTLA-4 gene, providing an A-G exchange. Moreover, we assessed the CTLA-4 49 (Thr/Ala) polymorphism in diabetic patients and first-degree relatives as compared to control subjects.

*Research design and methods*: Three loci (HLA-DQB1, DQA1 and CTLA-4) were analysed in 62 type 1 diabetic patients, 72 firstdegree relatives and 84 nondiabetic control subjects by means of PCR-RFLP.

*Results*: A significant enrichment in DQB1 alleles encoding for an amino acid different from Asp in position 57 (NA) and DQA1 alleles encoding for Arg in position 52 was observed in diabetic subjects and first-degree relatives as compared to controls. The genotype and allele frequencies of these polymorphisms in type 1 diabetic patients and firstdegree relatives differed significantly from those of controls (p< 0.001 and 0.05 respectively). CTLA-49 Ala alleles frequencies were 75.8% in type 1 diabetic patients and 68.1% in first-degree relatives in comparison to 35.7% in control subjects. The Ala/Ala genotype conferred a relative risk of 18.8 (p < 0.001).

*Conclusion*: The CTLA-4 49 Ala allele confers an increased risk of type 1 diabetes, independent of age and HLA-DQ genetic markers.


*Int. Jnl. Experimental Diab. Res.*, 3:1-9, 2002
© 2002 Taylor and Francis
1560-4284/02 $12.00 + .00

### Markers for Risk of Type 1 Diabetes in Relatives of Alsacian Patients with Type 1 Diabetes

JEAN CLAUDE ONGAGNA[a*], REMI SAPIN[b], MICHEL PINGET[a] AND ALAIN BELCOURT[a]

[a]Centre Européen d'Etude du Diabète-Hôpitaux Universitaires de Strasbourg

[b]Institut de Physique Biologique, Unité d'Analyses Endocriniennes UPRES-A 7004-ULP/CNRS, Faculté de Médecine, Université Louis Pasteur de Strasbourg

*Received: February 8, 2001; In final form: October 12, 2001*

*Background*: The cytotoxic T lymphocyte-associated antigen 4 gene (CTLA-4) encode the T cell receptor involved in the control of T cell proliferation and mediates T cell apoptosis. The receptor protein is a specific T lymphocyte surface antigen that is detected on cells only after antigen presentation. Thus, CTLA-4 is directly involved in both immune and autoimmune responses and may be involved in the pathogenesis of multiple T cell-mediated autoimmune disorders. There is polymorphism at position 49 in exon 1 of the CTLA-4 gene, providing an A-G exchange. Moreover, we assessed the CTLA-4 49 (Thr/Ala) polymorphism in diabetic patients and first-degree relatives as compared to control subjects.

*Research design and methods*: Three loci (HLA-DQB1, DQA1 and CTLA-4) were analysed in 62 type 1 diabetic patients, 72 first-degree relatives and 84 nondiabetic control subjects by means of PCR-RFLP.

*Results*: A significant enrichment in DQB1 alleles encoding for an amino acid different from Asp in position 57 (NA) and DQA1 alleles encoding for Arg in position 52 was observed in diabetic subjects and first-degree relatives as compared to controls. The genotype and allele frequencies of these polymorphisms in type 1 diabetic patients and first-degree relatives differed significantly from those of controls ($p< 0.001$ and 0.05 respectively). CTLA-49 Ala alleles frequencies were 75.8% in type 1 diabetic patients and 68.1% in first-degree relatives in comparison to 35.7% in control subjects. The Ala/Ala genotype conferred a relative risk of 18.8 ($p < 0.001$).

*Corresponding author: tel. (0) 33 88 11 54 67; fax (0) 33 88 11 63 52; e-mail : jean-claude.ongagna@wanadoo.fr

*Conclusion*: **The CTLA-4 49 Ala allele confers an increased risk of type 1 diabetes, independent of age and HLA-DQ genetic markers.**

*Keywords*: HLA-DQ; CTLA-4 gene, type 1 diabetes, first-degree relatives

### INTRODUCTION

Type 1 diabetes, is a chronic disease characterized by the autoimmune destruction of pancreatic β-cells resulting in complete insulin deficiency [1]. The disease is quite heterogeneous in its clinical expression [2]. A large body of evidence indicates that inherited genetic factors influence both susceptibility and resistance to the disease. There is significant familial clustering with an average prevalence of 6% in siblings as compared to 0.3% in a French Caucasian population. The major susceptibility locus lies within the HLA region on the short arm of chromosome 6 [3], and provides up to 40-50% of the inheritable risk [4]. An association between HLA class I alleles and type 1 diabetes was first described in the early 1970s [5, 6]. Subsequently, a closer association was demonstrated between HLA-DR alleles and type 1 diabetes [7] and DR3 and DR4 were shown to be the major alleles associated with this disease. More recent observations indicated that the genes in the HLA-DQ region were even more closely associated with type 1 diabetes than the DR genes [8, 9, 10]. Several studies confirmed the association of DQB1 genes with type 1 diabetes and demonstrated that DQB1*02/0302 combination was the genotype most closely associated with the disease.

Whole-genome searches have identified at least 15 additional chromosomal regions with putative linkage to type 1 diabetes [11]. So far only a minority of these candidate regions have been confirmed as true susceptibility loci in independent linkage and/or association studies. Recently, linkage to and/or association with type 1 diabetes of an A-to-G transition polymorphism at position 49 in the first exon of the CTLA-4-gene (cytotoxic T lymphocyte associated antigen-4) on chromosome 2q33 (designated IDDM12) have been shown in data sets from several countries [12]. This chromosomal region contains the CTLA4 and CD28 genes encoding for two molecules intimately involved in the regulation of T-cell activation and proliferation. Differential regulation of these mole-

TABLE 1 Primers and restriction enzymes used for PCR-RFLP methods for HLA-DQB1, HLA-DQA1 and CTLA-4.

| Primers | restriction enzymes | Location | Target (bp) | Expect bands (bp) |
|---|---|---|---|---|
| 5’-GATTTCGTGTACCAGTTTAAG-3’ | Acy I | HLA-DQB1 | | 241, 137, 104, 70 |
| 5’-CCACCTCGTAGTTGTGTCTGC-3’ | Hae III | exon 2 | 241 | 241, 150, 127, 95 |
| | Hha I | | | 189, 141, 112, 90 |
| | Hpa II | | | 241, 198, 126, 115 |
| 5’-GGTGTAAACTTGTACCAG-3’ | Dde I | HLA-DQA1 | | 225, 127, 118, 113 |
| 5’-GGTAGCAGCGGTAGAGTTG-3’ | Fok I | exon 2 | 225 | 225, 187 |
| | Rsa I | | | 225, 186, 183 |
| 5’-GCTCTACTTCCTGAAGACCT-3’ | Bb*v*-I | CTLA-4 | 162 | 162, 88, 74 |
| 5’-AGTCTCACTCACCTTTGCAG-3’ | | exon 1 | | |

TABLE 2 Prevalence of genetic markers in type 1 diabetic patients, first-degree relatives and control subjects.

| Genetic marker | Control subjects (n = 84) | Type 1 diabetic patients (n = 62) | First-degree relatives (n = 72) | RR |
|---|---|---|---|---|
| **HLA-DQB1** | | | | |
| NA 02 | 15 (8.9) | 52 (41.9)*** | 34 (23.6)*** | 7.5 |
| NA 0302 | 21 (12.5) | 38 (30.6)*** | 21 (14.6)* | 3.1 |
| Others NA | 12 (7.1) | 22 (17.7)§ | 33 (22.9) *** | 2.8 |
| A 0301 | 45 (26.8) | 7 (5.6)*** | 27 (18.8)* | 0.2 |
| A 0602 | 36 (21.4) | 0 (0) | 19 (13.2) ** | - |
| Others A | 39 (23.2) | 5 (8.1)*** | 10 (6.9) *** | 0.1 |
| NA/NA | 6 (7.1) | 47 (75.8)*** | 20 (27.8) *** | 40.7 |
| NA/A | 48 (57.1) | 15 (24.2)*** | 43 (59.7) ** | 0.2 |
| A/A | 30 (35.7) | 0 (0) | 9 (12.5) *** | - |
| **HLA-DQA1** | | | | |
| Arg 0301 | 24 (14.3) | 48 (38.7)*** | 38 (26.4) ** | 3.7 |
| Arg 0501 | 36 (21.4) | 46 (37.1)§ | 52 (36.1) ** | 2.2 |
| Non-Arg 01 | 68 (40.5) | 19 (15.3)*** | 42 (29.2) ** | 0.3 |
| Non-Arg 0201 | 30 (17.9) | 4 (3.2)*** | 8 (5.5) *** | 0.2 |
| Others | 10 (5.9) | 7 (5.6) | 4 (2.8) * | 0.9 |
| Arg/Arg | 23 (27.4) | 39 (62.9)*** | 25 (34.7) * | 4.5 |
| Arg/Non-Arg | 12 (14.3) | 20 (32.3)** | 36 (50.0) *** | 2.9 |
| Non-Arg/Non-Arg | 49 (58.4) | 3 (4.8)*** | 11 (15.3) *** | 0.04 |
| **CTLA-4** | | | | |
| Thr | 08 (64.3) | 30 (24.2)*** | 46 (31.9) *** | 0.2 |
| Ala | 60 (35.7) | 94 (75.8)*** | 98 (68.1) *** | 5.6 |
| Thr/Thr | 43 (51.2) | 3 (4.8)*** | 13 (18.1) *** | 0.05 |
| Thr/Ala | 27 (32.1) | 10 (16.1)** | 25 (34.7) * | 0.4 |
| Ala/Ala | 14 (16.7) | 49 (79.0)*** | 34 (47.2) *** | 18.8 |

Data are n (%). *$p > 0.05$; **$p < 0.05$; §$p < 0.01$; ***$p < 0.001$ compared with control subjects.

cules could easily affect T-cell function and hence the regulation of the immune response. Therefore, CTLA-4 gene is a strong candidate gene for autoimmune diseases including type 1 diabetes. The present study aims to examine the presence of genetic markers in a group of first degree relatives recruited during the same period as their diabetic probands.

### RESEARCH DESIGN AND METHODS

### *Patient and control subjects*

Seventy-two (29 males, 43 females) healthy first-degree relatives of type 1 diabetic patients (n = 62) from 25 families participating in the prospective based family study conducted in the province of Alsace (France) were analysed to investigate the role of genetic and environmental factors in the development of type 1 diabetes. Out of the diabetic patients, 34 were males and 11 had long-term type 1 diabetes of over 30 years without complications. Type 1 diabetes was diagnosed according to the WHO criteria [13]. The mean age at diabetes onset was 13.3 years (range 5-38), and the mean duration of the disease was 32.2 years (range 1-37). Their first-degree relatives aged below 45 years were invited to take part. Informed consent was obtained from the subjects. The local ethical committee approved the study design. Out of the first-degree relatives, 23 were siblings (age 20 ± 14), 34 were offsprings (age 19 ± 12), and 15 were parents (age 37 ± 6). Five (offspring) were GAD antibodies positive and 7

TABLE 3 Prevalence of genetic markers according to age at clinical onset of type 1 diabetes

| Genetic marker | Controls (n = 84) | Age at clinical onset (years) 0-9 (n = 14) | 10-19 (n = 19) | 20-39 (n = 29) | p |
|---|---|---|---|---|---|
| **HLA-DQB1** | | | | | |
| *02 | 15 (8.9) | 16 (57.1) | 18 (47.4) | 18 (31.0) | < 0.05 |
| *0302 | 21 (12.5) | 11 (39.3) | 14 (36.8) | 13 (22.4) | < 0.05 |
| **HLA-DQA1** | | | | | |
| *0301 | 45 (26.8) | 15 (53.6) | 18 (47.4) | 15 (25.9) | = 0.02 |
| *0501 | 36 (21.4) | 13 (46.4) | 16 (42.1) | 17 (29.3) | ns |
| **CTLA-4** | | | | | |
| Thr | 108 (64.3) | 7 (25.0) | 12 (31.6) | 11 (18.9) | ns |
| Ala | 60 (35.7) | 21 (75.0) | 26 (94.7) | 47 (56.9) | ns |
| Thr/Thr | 43 (51.2) | 1 (7.1) | 2 (10.5) | 0 (0) | ns |
| Thr/Ala | 27 (32.1) | 2 (14.3) | 5 (26.3) | 3 (10.3) | ns |
| Ala/Ala | 14 (16.7) | 11 (78.6) | 12 (63.2) | 26 (89.7) | ns |

Data are n (%), ns = not significant

(3 sibling and 4 offspring) were IA-2 antibodies positive. The group of control subjects were 84 randomly selected among normal blood donors matched for age, gender and geographically, all without known personal or familial history of diabetes or other autoimmune diseases.

### *PCR-RFLP*

The genomic DNA was subjected to PCR to amplify the polymorphic regions in exon 2 of the HLA-DQB1 gene (241 bp), DQA1 gene (225 bp) and exon 1 of the CTLA-4 gene (162 bp) as follows: 200 ng of genomic DNA was mixed with 20 µg of both upstream and downstream primers (Table 1), 10 mM dNTP, and 2.5 U of *Taq* polymerase in 50 µl of reaction mixture containing PCR buffer (10 mM Tris, pH = 8.4, 50 mM KCL, and 1.5 mM $MgCl_2$). Denaturation, annealing, and extension reactions were performed at 95°C for 1 min, 55°C for 2 min, and 72°C for 1 min respectively, and the amplification was continued for 29 cycles in a DNA Thermal Cycler (Mini Cycler, MJ Research, CA, USA). Ten of the amplified PCR products thus obtained were digested in 50 µl with 2.5 U of the restriction enzymes (Table 1). The fragments were analysed by PAGE.

### *Statistical analysis*

Differences in the prevalence of alleles or genotypes between diabetic, first-degree relatives and nondiabetic subjects were examined using a $\chi^2$ test with Yates' correction when appropriate (one expected number < 5). Statistical significance was defined as $p < 0.05$. The relative risk (RR) conferred by a given genetic marker was expressed as an odds ratio.

TABLE 4 Interaction between CTLA-4 polymorphism and HLA-DQ

| | | CTLA-4 alleles | | CTLA-4 genotypes | | |
|---|---|---|---|---|---|---|
| | n | Thr | Ala | Thr/Thr | Thr/Ala | Ala/Ala |
| Control subjects | 84 | 108 (64.3) | 60 (35.7) | 43 (51.2) | 27 (32.1) | 14 (16.7) |
| Type 1 diabetic Patients | 62 | 30 (24.2) | 94 (75.8) | 3 (4.9) | 10 (16.1) | 49 (79.0) |
| **HLA-DQB1** | | | | | | |
| *02 (+) | 26 | 12 (23.1) | 40 (76.9) | 2 (7.7) | 4 (15.4) | 20 (76.9) |
| *02 (-) | 36 | 18 (25.0) | 54 (75.0) | 1(2.8) | 6 (16.7) | 29 (80.6) |
| p | | ns | ns | - | ns | ns |
| *0302 (+) | 19 | 8 (21.1) | 30 (78.9) | 0 (0) | 3 (15.8) | 16 (84.2) |
| *0302 (-) | 43 | 22 (25.6) | 64 (74.4) | 3 (6.9) | 7 (16.3) | 33 (76.7) |
| p | | ns | ns | - | ns | ns |
| **HLA-DQA1** | | | | | | |
| *0301 (+) | 24 | 10 (20.8) | 38 (79.2) | 1 (4.2) | 4 (16.7) | 19 (79.2) |
| *0301 (-) | 38 | 20 (26.3) | 56 (73.7) | 2 (5.3) | 6 (15.8) | 30 (78.9) |
| p | | ns | ns | - | ns | ns |
| *0501 (+) | 23 | 12 (26.1) | 34 (73.9) | 0 (0) | 5 (21.7) | 18 (78.3) |
| *0501 (-) | 39 | 18 (23.1) | 60 (76.9) | 3 (7.9) | 5 (12.8) | 31 (79.5) |
| p | | ns | ns | - | ns | ns |

Data are n (%)

### RESULTS

### *HLA-DQ* ALLELE AND GENOTYPE DISTRIBUTION

The analysis of PCR-RFLP amplified DNA from 62 type 1 diabetic patients, 72 first-degree relatives and 84 controls matched for age and sex revealed several DQB1 and DQA1 alleles (Table 2). Comparing controls and diabetic patients, a significant differences appeared between diabetic patients and healthy control subjects (Table 2). Indeed there is an increase in Non-Asp57 (NA) allele from 28.6% in controls to 90.3% in diabetics ($p < 0.001$ ; $\chi^2 = 109$ ; OR = 23.3). In contrast, Asp 57 (A) allele showed a decrease from 71.4% in controls to 9.7% in diabetic patients ($p < 0.001$ : $\chi^2 = 110$ ; OR = 0.04). Moreover, the NA/A ratio was reversed between controls (0.4) and diabetics (9.2). Finally, an intermediate pattern was observed for these alleles in the first degree relatives (Table 2). Out of the 72 first-degree relatives, 27.8% (20/72) were homozygous NA/NA, 59.7% (43/72) were heterozygous NA/A, and 12.5% (9/72) were homozygous A/A. In controls, the frequency was 7.1% for NA/NA, 57.1% for NA/A and 35.7% for A/A. The HLA-DQB1*02 (NA) and DQB1*0302 (NA) were significantly increased in type 1 diabetic patients ($\chi^2 = 43$; $p<10^{-3}$) as compared to control subjects. The HLA-DQB1*02 allele was also significantly increased in first-degree relatives ($\chi^2 = 12.6$; $p < 10^{-3}$). The relative risk conferred by the HLA-DQB1*0302 allele was less determinant than DQB1*02 (RR = 3.1 and 7.5 respectively). Moreover, the DQA1*0301 (Arg52) and DQA1*0501 (Arg52) alleles were found highly increased in type 1 diabetics and first-degree relatives (Table 2). Conversely, the frequencies of HLA-DQB1*0602 (Asp57), DQA1*01 (Non-Arg52) and DQA1*0201 (Non-Arg52) alleles were decreased among patients and first-degree relatives.

### *CTLA-4 49 (A/G) polymorphism*

The genotype and gene frequencies of CTLA-4 polymorphism in type 1 diabetes and first-degree relatives differed significantly ($p < 10^{-3}$) from those of control subjects (Table 2). Frequencies in the base transition in codon 49 to encode for Thr or Ala were 24.2% (30/124) and 75.8% (94/124) respectively in patients with type 1 diabetes; 31.9% (46/144) and 68.1% (98/144) in first-degree relatives, whereas 64.3% (108/168) and 35.7% (60/168) were found in controls ($\chi^2 = 43.5$; $p < 10^{-3}$ and $\chi^2 = 33.5$; $p < 10^{-3}$ respectively). The frequency of the Ala/Ala genotype was increased in diabetics and first-degree relatives (79% and 47.2% respectively versus 16.7% in control subjects). The relative risk of type 1 diabetes conferred by the Ala/Ala genotype was rather high and moderate with the Ala allele (RR = 18.8; $10^{-3}$ and RR = 5.6; $10^{-3}$ respectively).

### *Prevalence of genetic markers according to age at clinical onset of type 1 diabetes*

The 62 patients were stratified according to age in three categories: 0-9 (n = 14), 10-19 (n = 19), and 20-39 (n = 29) years (Table 3). The fraction of patients carrying the Ala allele or Ala/Ala genotype did not differ significantly among the various groups. Consequently, the presence for the Thr allele or Thr/Thr and Thr/Ala genotypes was also age-independent as previously showed [25]. In contrast, within the same group, the prevalence of HLA-DQB1*0302 and DQA1*0301 alleles declined with age at onset.

### *CTLA-4 49 (A/G) polymorphism and HLA-DQ-linked risk*

Because the difference in HLA-DQ locus in diabetic subjects might affect the distribution of the CTLA-4 alleles or genotypes to susceptibility to diabetes, the patients were sub-divided according to HLA-DQ and comparison was made between the diabetic patients with or without HLA-DQB1*02, DQB1*0302, DQA1*0301 or DQA1*0501. However, the frequency of CTLA-4 alleles or genotypes was independent of the susceptible HLA-DQ status (Table 4).

### DISCUSSION

Susceptibility to type 1 diabetes has been previously shown to be highly correlated with the absence of an Asp57 on the DQβ chain [14], and the presence of an Arg52 on the DQα chain [15]. Nevertheless, the absence or presence of these residues in the DQ sequences of type 1 diabetic patients cannot entirely determine the susceptibility or the resistance to the disease [14, 16]. In this study we confirmed the importance of DQβAsp57 and DQαArg52 codons in that susceptibility. The distribution of HLA-DQB1 and DQA1 alleles among diabetic patients and healthy controls was comparable to that in previous reports and closely matched with the reported frequencies in Caucasian individuals [17, 18, 19]. The frequency of high-risk alleles DQB1*02, DQB1*0302 and DQA1*0301 increased in type 1 diabetic patients. We also confirmed the high frequency of HLA-DQ risk alleles in first-degree relatives [20, 21, 22].

Age-dependent HLA heterogeneity has been observed in Caucasian type 1 diabetic patients indicating that high risk HLA genotypes or alleles occurred at a higher frequency among the younger age onset groups [23, 24]. In this study, diabetic patients were stratified according to age of onset in three categories: 0-9, 10-19, and 20-39 years. Our data analysis of HLA-DQB1 alleles confirm this age-dependent susceptibility. The HLA-DQB1 alleles (DQB1*02 and DQB1*0302) were present in 53.4% of adults patients (20-39 yrs) and 96.4% in type 1 diabetics children (0-9 yrs) as compared to only 21.4% in controls (p<

0.001). Regarding the DQA1*0301 allele, there were also difference between the adult and younger patients. Previous studies in other countries already showed this HLA-age-dependent risk [25, 26]. Caillat-Zucman *et al.* found a decreased frequency of DR3 and DR4 haplotypes and DR3/DR4 heterozygosity amongst patients who had developed diabetes after the age of 15 [17].

Moreover, the probable involvement of CTLA-4 49 (Thr/Ala) polymorphism in type 1 diabetes was analysed in this study. Our results based on PCR-RFLP of exon 1 in 218 individuals, including 62 type 1 diabetic patients and 72 first-degree relatives, proved the involvement of a CTLA-4 49 (Thr/Ala) polymorphism in type 1 diabetes susceptibility, consistent with the data sets from several countries including Belgium, Germany and Japan [12, 27, 28, 29], but not in others [30]. Thus, there seems to be an ethnic difference in the role of the CTLA-4 49 (Thr/Ala) polymorphism. CTLA-4 is a member of the immunoglobulin superfamily, more specifically of the subgroup of molecules with a single V (variable) domain [31] and detectable on T cells only after antigen presentation. CTLA-4 deficient mice have shown extensive T cell proliferation, B cell activation, and a marked rise in serum immunoglobulin concentrations [32]. Signalling through CTLA-4 may down-regulate proliferative responses by inhibiting the production of IL-2, or the expression of IL-2 receptors [33, 34]. Therefore CTLA-4 plays an important inhibitory role in regulating lymphocyte expansion and might be responsible for the disruption of the delicate balance between TH1 and TH2 lymphocytes by preventing down-regulation of activated self reactive T cells [35].

In contrast to the HLA-DQB1 alleles, the risk conferred by CTLA-4 Ala allele did not decrease with age at clinical onset, confirming the results of Van der Auwera [25]. HLA locus may have strong confounding effects and obscure the role of CTLA-4 49 (Thr/Ala) polymorphism in a case-control study [36]. Thus, we further analysed this polymorphism in conjunction with DQ locus and failed to detect any difference in its effect according to HLA-DQ status like Van der Auwera [25]. It was found in this study that association of CTLA-4 with diabetes was not affected by HLA-DQ susceptibility alleles. This last result is in contradiction with Badenloop's data, which showed that the CTLA-4 predisposing variant increased diabetes risk in synergy with HLA-DR3 but not with HLA-DR4 in a German population [37]. The discrepancy between ours and the result of Badenloop *et al* [37] might be due to the size and degree of homogeneity of families. The size of ours families was much smaller than that of Badenhoop's German families (25 vs130).

The analysis of an A-G transition in the first exon of the CTLA-4 gene, coding for a Thr/Ala substitution in the leader peptide, showed a preferential transmission to the affected siblings [12]. A similar relationship was also reported in Japanese patients with type 1 diabetes and with autoimmune thyroid disease [28, 38]. Although, such a relationship was not observed in families from Sardinia, U.K, U.S.A, or Denmark [39, 40].

In conclusion, this study demonstrated that CTLA-4 49 (Thr/Ala) polymorphism was associated with type 1 diabetes and that the CTLA-4 49 Ala allele conferred an increased risk to type 1 diabetes independently of age and HLA-DQ genetic markers.

*ACKNOWLEDGEMENTS*

We thank M.C. KALTENBACHER and E. HOTMANN for their excellent technical assistance.

### REFERENCES

[1] Eisenbarth, G.S. (1986) Type I diabetes mellitus: a chronic autoimmune disease. N Engl J Med, **314**, 1360-1368.

[2] Seissler, J., de Sonnavelle, J. J., Morgenthaler, N. G., Steinbrenner, H., Glauve, D., Khoo-Morgenthaler, W. A. (1998) Immunological heterogeneity in type 1 diabetes: presence of distinct autoantibody patterns in patients with acute onset and slowly progressive disease. Diabetologia, **41**, 891-897.

[3] Johnson, A. H., Hurley, C. K., Hartzman, R. J., Alper, C. A., Yunis, E.J. (1991) HLA: The major histocompatibility complex of man. In: 18th edition Clinical Diagnosis & Management by Laboratory Methods. Henry, J. B., editor. 18th ed. Saunder, W. B., Philadelphia pp 761-794.

[4] Nobel, J. A., Valdes, A. M., Cook, M., Klitz, W., Thomson, G., Erlich, H. A. (1996) The role of HLA class II genes in insulin-dependent diabetes mellitus: molecular analysis of 180 Caucasians multiplex families. Am J Hum Genet, **59**, 1134-1148.

[5] Singal, D. P., Biajchman, M. A. (1973) Histocompatibitily (HLA) antigens, lymphocytotoxic antibodies and tissues antibodies in patients with diabetes mellitus. Diabetes, **22**, 429-432.

[6] Nerup, J., Platz, P., Andersen, O. O., Christy, M., Lyngsoe, J., Poulsen, J. E., Ryder, L. P., Nielsen, L. S., Thomsen, M., Svejgaard, A. (1974) HL-A antigens and diabetes mellitus. Lancet, **ii**, 864-866.

[7] Solow, H., Hidalgo, R., Singal, D. P. (1979) Juvenile-onset diabetes: HL-A, -B, -C, and DR alloantigens. Diabetes, **28**, 1-4.

[8] Owerbach, D., Lernmark, Å., Platz, P., Ryder, L. P., Rask, L., Peterson, P. A., Ludvigsson, J. (1983) HLA-D region b-chain DANN endonuclease fragments differ between HLA-DR identical healthy and insulin-dependent diabetic individuals. Nature, **303**, 815-817.

[9] Nepom, B. S., Palmer, J., Kim, S. J., Hansen, J. A., Holmbeck, S. L., Nepom, G. T. (1986) Specific genomic markers for the HLA-DQ subregion discriminate between DR4+ seropositive juvenile rheumatoid arthritis. J Exp Med, **164**, 345-350.

[10] Morel, P. A., Dorman, J. S., Todd, J. A., McDevitt, H. O., Trucco, M. (1988) Aspartic acid at position 57 of the HLA-DQb chain protects against type I diabetes: a family study. Proc Natl Sci USA, **85**, 8111-8115.

[11] Davis, J. L., Kawaguchi, Y., Bennett, S. T., Copeman, J. B., Codell, H. J., Pritchard, L. E., Reed, P. W., Gough, S. C. L., Jenkins, S. C., Palmer, S. M., Balfour, K. M., Rowe, B. R., Farral, M., Bennett, A. H., Bain, S. C., Todd, J. A. (1994) A genome-wide search for human type 1 diabetes susceptibility genes. Nature, **371**, 130-136.

[12] Nistico, L., Buzzetti, R., Pritchard, L. E., Van Der Auwera, B., Giovannini, C., Bosi, E., Larrad, M. T., Rios, M. S., Chow, C., Cockram, C. S., Jacobs, K., Mijovic, C., Bain, S. C., Barnett, A. H., Vandewalle, C. L., Schuit, F., Gorus, F. K., Belgian Diabetes Registry, Tosi, R., Pozzilli, P., Todd, J. A. (1996). The *CTLA-4* gene region of chromosome 2q33 is linked to, and associated with type 1 diabetes: Belgian Diabetes Registry. Human Molecular Genetics, **5**, 1075-1080.

[13] WHO (1985). Diabetes mellitus, report of a WHO study group. WHO technical report Geneva, 727.

[14] Todd, J. A., Bell, J. I., McDevitt, H. O. (1987) HLA-DQβ gene contributes to susceptibility and resistance to insulin-dependent diabetes mellitus. Nature, **329**, 599-604.

[15] Khalil, I., d'Auriol, L., Gobet, M., Morin, L., Lepage, V., Deschamps, I., Sik, Park, M., Degos, L., Galibert, F., Hors, J. (1990) Combination of HLA-DQβAsp57-negative and HLA-DQαArg52 confers susceptibility to insulin-dependent diabetes mellitus. J Clin Invest, **85**, 1315-1319.

[16] Owerbach, D., Gunns, T, G., Wible, L., Gabbay, K. A. (1988) Oligonucleotide probes for HLA-DA and DQB genes definie susceptibility to type 1 (insulin-dependent) diabetes mellitus. Diabetologia, **31**, 751-757.

[17] Caillat-Zucman, S., Garchon, H. J., Timsit, J., Assan, R., Boitard, C., Djilali-Saiah, I., Bourgnères, P., Bach, J. F. (1992) Age-dependent HLA genetic heterogeneity of type 1 insulin-dependent diabetes mellitus. J Clin Invest, **90**, 2242-2250.

[18] Kockum, I., Wassmuth, R., Holmberg, E., Michelsen, B., Lernmark, A. (1993) HLA-DQ primarily confers protection and HLA-DR susceptibility in type I (insulin-dependent) diabetes studied in population-based affected families and controls. Am J Hum Genet, **53**, 150-167.

[19] Ongagna, J. C., Kaltenbacher, M., C., Sapin, R., Pinget, M., Belcourt, A. (2000) The HLA-DQB alleles and amino acid variants of the vitamin D-binding protein in diabetic patients in Alsace. Clin Biochem, **34**, 59-63.

[20] Serrano-Rios, M., Gutierrez-Lopez, M. D., Perez-Bravo, F., Martinez, M. T., Antona, J., Rowley, M., Mackay, I., Zimmet, P. (1996) HLA-DR, DQ and anti-GAD antibodies in first-degree relatives of type 1 diabetes mellitus. Diabetes Res Clin Pract, **34**, Suppl: S33-39.

[21] Pugliesse, A., Gianani, R., Moromisato, R., Awdeh, Z. L., Alper, C. A., Erlich, H., A., Jackson, R. A., Eisenbarth, G. S. (1995) HLA-DQB1*0602 is associated with dominant protection from diabetes even among islet cell antibody positive first-degree relatives of patients with insulin-dependent diabetes. Diabetes, **44**, 608-613.

[22] Kulmala, P., Savola, K., Reijonen, H., Veijola, R., Vähäsalo, P., Karjalainen, J., Tuomilehto-Wolf, E., Ilonen, J., Tuomilehto, J., Åkerblom, H. K., Knip, M., and the Childhood Diabetes in Finland Study Group. (2000) Genetic Markers, Humoral Autoimmunity, and Prediction of Type 1 Diabetes in Siblings of Affected Children. Diabetes, **49**, 48-58.

[23] Karjalainen, J., Salmela, P., Ilonen, J., Surcil, H. M., Knip, M. (1989) A comparison of childhood and adult type I diabetes mellitus. N Engl J Med, **320** (14), 881-886.

[24] Valdes, A. M., Thomson, G., Erlich, H. A., Noble, J. A. (1999) Association between type 1 diabetes, age of onset, and HLA among sibling pairs. Diabetes, **48**, 1658-1661.

[25] Van Der Auwera, B. J., Vandewalle, C. L., Schuit, F. C., Winnock, F., De Leeuw, I. H., Van Imschoot, S., Lamberigts, G., Gorus, F. K., Belgian Diabetes Registry. (1997) CTLA-4 gene polymorphism confers susceptibility to insulin-dependent diabetes mellitus (IDDM) independently from age and from other genetic or immune disease markers. Immunol, **110**, 98-103.

[26] Thomson, G., Robinson, W. P., Kuhner, M. K., Joe, S., MacDonald, M. J., Gottschall, J. L., Barbosa, J., Rich, S. S., Berttram, J., Baur , M. P. (1988) Genetic heterogeneity, mode of inheritance, and risk estimate for a joint study of Caucasians with insulin-dependent diabetes mellitus. Am J Hum Genet, **43**, 799-816.

[27] Donner, H., R, H., Walfish, P.G., Braun, J., Siegmund, T.,

Finke, R., Herwig, J., Usadel, K. & Badenhoop, K. (1997). CTLA-4 Alanine-17 confers genetic susceptibility to Graves' disease and to type 1 diabetes mellitus. J of Clin Endocrinand Metab, **82**, 143-146.

[28] Awata, T., Kuruhara, S., Litaka, M., Takei, S. I., Inoe, I., Ishii, C., Negishi, K., Izumida, T., Yoshida, Y., Hagura, R., Kuzuya, N., Kanazawa, Y., Katayama, S. (1998) Association of CTLA-4 gene A-G polymorphism (IDDM12 locus) with acute-onset and insulin-depleted IDDM as well as autoimmune thyroid disease (Graves' disease and Hashimoto's thyroiditis) in the Japanese population. Diabetes, **47**, 128-129.

[29] Djilali-Saiah, I., Larger, E., Harfouch-Hammound, E., Timsit, J., Clere, J., Bertin, E., Assan, R., Boitard, C., Bach, J. F., & Caillat-Zucman, S. (1998). No major role for the CTLA-4 gene in the association of autoimmune thyroid disease with IDDM. Diabetes, **47**, 125-127.

[30] Marron, H.P., Raffel, L. J., Jacob, C. O., Serrano-Rios, M., Martinez Larrad, M. T., Teng, W. P., Park, Y., Zhang, Z-X., Goldstein, D. R., Tao, Y-W., Beaurain, G., Bach, J-F., Huang, H-S., Luo, D-F., Zeidler, A., Rotter, J. L., Yan, M. C. K., Modilevsky, T., Maclaren, N. K., She, J-X. (1997). Insulin-dependent diabetes mellitus (IDDM) is associated with CTLA-4 polymorphism in multiple ethnic groups. Human Molecular Genetics, **6**, 1275-1282.

[31] Harper, K., Balzano, C., Rouvier, E., Mattei, M. G., Luciani, M. F., Golstein, P. (1991) CTLA-4 and CD28 activated lymphocyte molecules are closely related in both mouse and human as to sequence, message expression, gene structure, and chromosomal location. J of Immun, **147**, 1037-1044.

[32] Waterhouse, P., Penninger, J. M., Timms, E., Wakeham, A., Shahinian, A., Lee, K. P., Thompson, C. B., Griesser, H., Mak, T. W. (1995). Lymphoproliferative disorder with early lethality in mice deficient in CTLA-4. Science, **270**, 985-988.

[33] Krummel, M. F., Allison, J. P. (1996) CTLA-4 engagement inhibits accumulation and cell cycle progression upon activation of resting T cell. J Exp Med, **183**, 2533-2540.

[34] Walunas, T. L., Bakker, C Y., Bluestone, J. A. (1996). CTLA-4 ligation blocks CD28-dependent T-cell activation. J Exp Med, **183**, 2541-2550

[35] Tivol, E. A., Borullo, F., Schweitzer, A. N., Lynch, W. P., Bluestone, J. A., Sharpe, A. H. (1995). Loss of *CTLA-4* leads to massive lymphoproliferation and fatal multiorgan tissue destruction, revealing a critical negative regulatory role of *CTLA-4*. Immunity, **3**, 541-547.

[36] Owerbach, D., Naya, F. J., Tsai, M., Allander, S. V., Powell, D. R., Gabbay, K. H. (1997) Analysis of candidate genes for susceptibility to type 1 diabetes. A case-control and family-association study of genes on chromosome 2q31-35. Diab, **46**, 1069-1074.

[37] Badenloop, K., Donner, H., Pani, M., Rau, H., Siegmund, T., Braum, J. (1999) Genetic susceptibility to type 1 diabetes: Clinical and molecular heterogeneity of IDDM1 and IDDM12 in a German population. Exp Clin Endocr Diabetes, **107** (3), S89-S92.

[38] Hayashi, H., Kusuka, I., Nagasaka, S., Kawakami, A., Rokkaku, K., Nakamura, T., Saito, T., Higashiyama, M., Honda, K., Ishikawa, S-E., Saito, T. (1999). Association of CTLA-4 polymorphism with positive anti-GAD antibody in Japanese subjects with type 1 diabetes mellitus. Clin Endocr, **51**, 793-799.

[39] Yanagawa, T., Maruyama, T., Gomi, K., Taniyama, M., Kasuga, A., Ozuwa, Y., Terauchi, M., Hirose, H., Maruyama, H., Saruta, T. (1999) Lack of association between CTLA-4 gene polymorphism and IDDM in Japanese subjects. Autoimmunity, **29**, 53-56.

[40] Larsen, Z. M., Kristiansen, O. P., Mato, E., Johannsen, J., Puig-Domingo, M., de Leiva, A., Nerup, J., Pociot, F. (1999) IDDM12 (CTLA-4) on 2q33 and IDDM13 on 2q34 in genetic susceptibility to type 1 diabetes (insulin-dependent). Autoimmunity, **31**, 35-42.

